# Methods matter: the influence of method on infection estimates of the bumblebee parasite *Crithidia bombi*

**DOI:** 10.1017/S0031182023001002

**Published:** 2023-11

**Authors:** Hannah S. Wolmuth-Gordon, Anisah Sharmin, Mark J. F. Brown

**Affiliations:** Royal Holloway University of London, UK

**Keywords:** epidemiology, experimental design, measuring infection, pollinator, trypanosome

## Abstract

The bumblebee gut parasite, *Crithidia bombi*, is widespread and prevalent in the field. Its interaction with *Bombus* spp. is a well-established epidemiological model. It is spread faecal-orally between colonies *via* the shared use of flowers when foraging. Accurately measuring the level of infection in bumblebees is important for assessing its distribution in the field, and also when conducting epidemiological experiments. Studies generally use 1 of 2 methods for measuring infection. One approach measures infection in faeces whereas the other method measures infection in guts. We tested whether the method of measuring infection affected the estimation of infection. Bumblebees were inoculated with a standardized inoculum and infection was measured 1 week later using either the faecal or gut method. We found that when the gut method was used to measure infection intensity estimates were significantly different to and approximately double those from the faecal method. These results have implications for the interpretation of previous study results and for the planning of future studies. Given the importance of bumblebees as pollinators, the impact of *C. bombi* on bumblebee health, and its use as an epidemiological model, we call on researchers to move towards consistent quantification of infections to enable future comparisons and meta-analyses of studies.

## Graphical abstract

**Figure:**
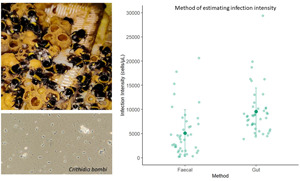

## Introduction

*Crithidia bombi* is a widespread and prevalent gut parasite of bumblebees (e.g. Shykoff and Schmid-Hempel, 1991; Rutrecht and Brown, 2008; Gillespie, 2010; Popp *et al*., 2012) and is transmitted faecal-orally between colonies *via* the shared use of flowers by foragers (Durrer and Schmid-Hempel, 1994; Graystock *et al*., 2015; Adler *et al*., 2018; Figueroa *et al*., 2019; Pinilla-Gallego *et al*., 2022) and within colonies *via* contact between infected individuals, faeces and contaminated surfaces (Schmid-Hempel and Schmid-Hempel, 1993; Otterstatter and Thomson, 2007; Sah *et al*., 2021). The interaction between *Bombus* spp. and *C. bombi* is easily manipulated and maintained in the laboratory, making it a well-established epidemiological model (Schmid-Hempel *et al*., 2019). Consequently, accurate measurement of the prevalence and intensity of *C. bombi* infections in bumblebees is important both for assessing infection levels in wild populations and when testing and investigating epidemiological questions.

There are 2 commonly used methods to measure the prevalence and intensity of infection; hereafter referred to as the faecal (e.g. Schmid-Hempel and Schmid-Hempel, 1993; Brown *et al*., 2000; Vaughan *et al*., 2022) and gut sampling method (e.g. Anthony *et al*., 2015; Biller *et al*., 2015; LoCascio et al., 2019*a*, 2019*b*) (see Table S1). To measure infection using the faecal sampling method, a faecal sample is taken and viewed under the microscope immediately, and *C. bombi* cells are counted. To measure infection using the gut sampling method, the mid- and hindguts are removed (this can be from a newly dead or frozen bee) and finely ground in 25% Ringer solution, left to settle for 3–5 h at room temperature and the supernatant is sampled and viewed under the microscope to identify *C. bombi* cells. Previously, the faecal sampling method has been compared to a similar, but not identical, gut sampling method. In this gut sampling method (hereafter referred to as gut sampling method 2) samples are left to settle for 24 h at 5°C (Otterstatter and Thomson, 2006). Otterstatter and Thomson (2006) used the faecal method and gut sampling method 2 to produce 2 measures of infection intensity for individual bees, but without controlling for inoculation dose. This showed that the 2 measures of infection intensity were positively correlated, with faecal counts giving higher intensity than gut counts. This is surprising, as one might expect that the gut sampling method 2 is more sensitive to detecting infections as *C. bombi* replicates in the gut prior to shedding cells in the faeces (Logan *et al*., 2005) and continues to do so throughout the infection. Furthermore, Otterstatter and Thomson (2006) estimated infection intensity using the faecal and gut sampling methods on the same individual. Faecal counts must always be taken before gut counts and therefore, the individual always defaecated and cleared a large number of *C. bombi* cells prior to gut sampling. Consequently, the infection intensity estimates obtained *via* gut sampling in this study are likely underestimates.

Whether the 2 methods described above (faecal and gut sampling method) yield similar results has implications for the interpretation of the results of previous studies. If 1 method is more sensitive at detecting infection, prevalence estimates will be higher, because the method will be more likely to detect low intensity infections. Estimates of infection intensity will also be higher, because a larger number of cells will be counted. In addition, knowledge of the comparability of the 2 methods can inform the design of future studies, for example if low infection intensity is expected the more sensitive method may be suitable. Here, we investigated whether the 2 methods produce similar estimates of infection by inoculating bumblebees with a standardized *C. bombi* dose and measuring the prevalence and infection intensity in bees 1 week later using 1 of 2 methods. We hypothesized that the gut sampling method would be a more sensitive measure of infection, leading to higher estimates of prevalence and infection intensity. This is because the gut is where the parasite is replicating and although Otterstatter and Thomson (2006) found higher estimates of infection intensity using faecal sampling compared to gut sampling, both measurements were taken on the same individual with faecal sampling always conducted prior to gut sampling.

## Materials and methods

### Experimental organisms

A total of 3 *Bombus terrestris audax* colonies, of 85–100 workers each, were ordered from Agralan (UK). Colonies were housed under red light at approximately 25°C and ambient humidity (49–54% relative humidity). They were fed sterile sugar solution (50% concentration) and honeybee collected pollen (Agralan, UK) *ad-libitum*. Upon arrival, the faeces of 10 individuals per colony were viewed under a phase-contrast microscope (Nikon Eclipse 50i) at ×400 and screened for *C. bombi, Apicystis bombi and Vairimorpha bombi.* All individuals were free of infection.

*Crithidia bombi* was obtained from 2 laboratory colonies of *Bombus terrestris audax* (Agralan, UK) that were infected and maintained for *C. bombi* stock. The parasite originated from post-hibernation spring queens of *B. terrestris audax* caught in Windsor Great Park (Surrey, UK) in March 2021 and March 2022, since when it has been continually cycled through laboratory colonies (Agralan, UK).

### Experimental design

#### Treatments

To investigate whether the measurement method affected the estimate of prevalence and infection intensity bees were infected with a standardized dose of *C. bombi.* Their infection intensity was measured 1 week later *via* either the faecal sampling or gut sampling method (for a comparison of papers using each method see Table S1). Inoculations occurred in 3 blocks over 3 days. On each day, 5 individuals from each of the 3 colonies were inoculated, resulting in 45 individuals in each method treatment group.

#### Inoculation

Bees were inoculated with a dose of 20 000 cells to test whether measurement method affected both prevalence (the number of infected individuals out of the number inoculated) and infection intensity (the number of *C. bombi cells* per *μ*L of sampled faeces or gut). Ruiz-González and Brown (2006) trialled a range of inoculation doses and 20 000 cells resulted in ~90% of individuals getting infected. A 90% infection rate yields a sufficient sample size to allow the investigation of infection intensity, whilst the fact that not all individuals get infected enables prevalence to be investigated. In addition, this dose is field-realistic (Schmid-Hempel and Schmid-Hempel, 1993).

On the day of inoculation, bees were removed from their colonies and weighed in pre-weighed vials to the nearest milligram (Scout SKX, Ohaus, Switzerland). Infection intensity can covary with size and therefore, mass was used as a proxy for size (Otterstatter and Thomson, 2006). Mass was used, rather than inter-tegular distance or wing marginal cell length, due to time constraints on inoculation days. We appreciate that body mass may be influenced by sugar consumption, but as all bees had equal exposure to *ad-libitum* food prior to weighing, this seems unlikely to have a meaningful impact on results. Bees were housed in nicot cages (Becky's bees, UK), which are cylindrical containers adapted from hair rollers to house bees (see Fig. S1). Bees were starved for 2 h prior to inoculation, to increase consumption of the inoculum. Faeces were collected from 20 individuals per *C. bombi* stock colony and purified using a modified triangulation protocol (Cole, 1970; Baron *et al*., 2014). Cell concentration of all *C. bombi* lifecycle stages (amastigote, choanomastigote, promastigote; Logan *et al*. (2005)) was calculated by viewing the purified faeces using an improved Neubauer haemocytometer under a phase-contrast microscope (Nikon Eclipse 50i) at ×400. Purified faeces were then mixed with sterile sugar solution (50% concentration) to produce a cell concentration of 667 cells/*μ*L that would yield a dose of 20 000 cells in 30 *μ*L. Bees were given a 30 *μ*L droplet of inoculum in a 2 mL syringe which was attached to the base of the nicot cage with masking tape. Bees were left to drink the inoculum for 4 h. Only individuals that had consumed the entire inoculum were included in the experiment. Bees remained housed in nicot cages for 1 week. 2 mL syringes were replaced with 5 mL syringes containing sterile sugar solution (50% concentration) and these were replaced every 3 days to prevent fungal growth.

#### Measuring infection

At 1 week after inoculation prevalence and infection intensity were measured. Bees in the faecal sampling treatment group were removed from their nicot cages and immediately put in specimen tubes containing 10 *μ*L microcapillary tubes to collect the faeces. The majority of bees defaecated within 0–30 min after being put in the specimen tubes. Faeces were viewed under the microscope immediately or as soon as possible depending on the number of bees that defecated simultaneously. Faeces were viewed on an improved Neubauer haemocytometer under a phase-contrast microscope at ×400 magnification and the prevalence and infection intensity were recorded. We counted all *C. bombi* lifecycle stages (amastigote, choanomastigote, promastigote; Logan *et al*. (2005)). Bees in the gut dissection treatment group were frozen at −80°C, to be dissected at a later date. On the day of dissection, bees were removed from the freezer and carefully held for 30 seconds to defrost. The crop, mid- and hindguts were removed from the bee. The mid- and hindguts were put in a 2 mL Eppendorf with 300 *μ*L of 25% Ringer solution (Ohaus, Thermo Scientific, UK). The guts were ground vigorously with a pestle for 30 s to release the *C. bombi* from the guts. The mixture was vortexed for 10 s and left to stand for 3 h to allow gut debris to sink to the bottom and a supernatant to form. A time duration of 3 h was chosen because previous studies have used this settling time (e.g. Anthony *et al*., 2015; LoCascio, Pasquale, *et al*., 2019*b*) and this was feasible in an experimental day, given the number of bees being screened for infection. After 3 hours, 10 *μ*L of the supernatant was viewed on an improved Neubauer haemocytometer under a phase-contrast microscope at ×400 magnification and the prevalence and infection intensity were recorded. Again, we counted all *C. bombi* lifecycle stages (amastigote, choanomastigote, promastigote; Logan *et al*. (2005)).

### Statistical analyses

Analyses were performed in RStudio ‘Prairie Trillium’ (RStudio Team, 2022), R version 4.2.0 (R Core Team, 2022). All figures were created using the ggplot() function from the ggplot2 package (Wickham, 2016). The effect of measurement method on prevalence of infection could not be tested since all individuals were infected. To test whether the measurement method affected the estimate of infection intensity a general linear model with a negative binomial error distribution and a log link was used due to overdispersion. The function ‘glm.nb’ was used from the MASS package (Venables and Ripley, 2002) with cells per microlitre as the response variable. The full model included method as a fixed factor and bee mass, colony and experimental block as covariates. We did not test a sufficient number of colonies to include colony as a random effect in a mixed effects model (Gelman and Hill, 2006; Arnqvist, 2020). Overdispersion was checked using the performance package (Lüdecke *et al*., 2021) and residuals with the DHARMa package (Hartig, 2022). The full and reduced models were compared using a likelihood ratio Chi-squared test. These values and AIC values were used to compare the reduced and full models.

## Results

**Figure 1.**
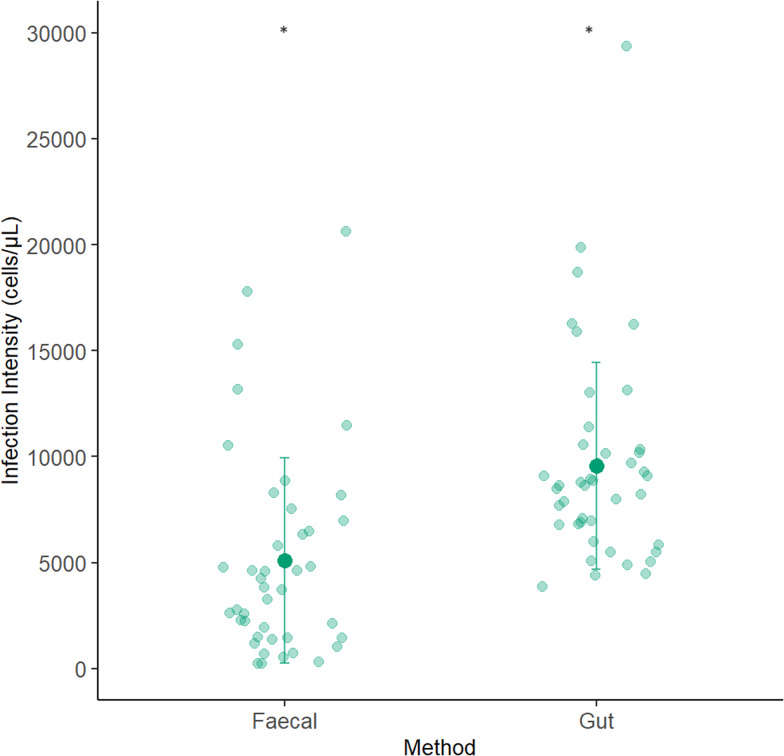
Infection intensity estimate using each method. The infection intensity of *C. bombi* 1 week after bees were given a standardized dose. Infection intensity was measured using 2 methods (faecal or gut sampling methods). There was a significant difference between the infection intensity estimates obtained *via* the faecal and gut sampling method. The large, darker datapoints show the mean infection intensity and the bars the standard deviations. Light datapoints show the raw data.

**Figure 2.**
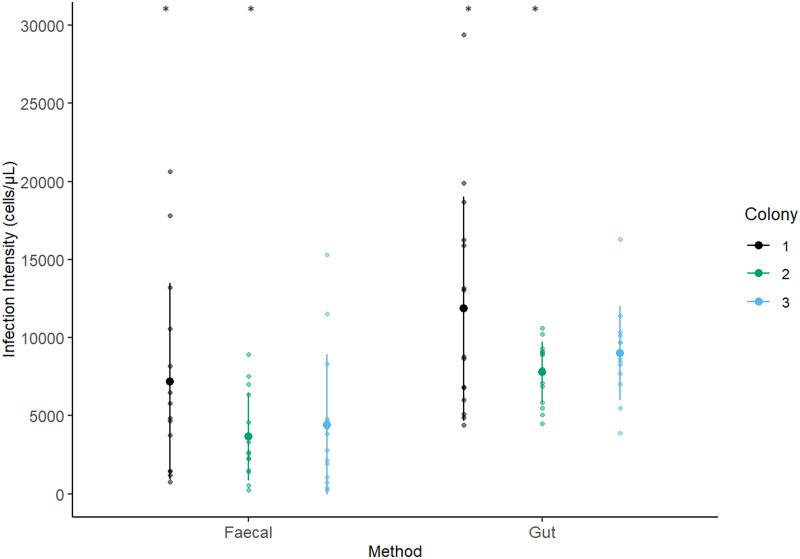
Infection intensity using each method separated by colony. The infection intensity of *C. bombi* in *B. terrestris* 1 week after a standardized inoculation dose. Infection intensity was measured using either a faecal or gut sampling method. Colony 1 had significantly higher infection intensity across both methods than colony 2. Colour indicates bee colony (black: colony 1, green: colony 2 and blue: colony 3). Smaller datapoints are the raw datapoints and mean infection intensity is shown by the larger datapoints. Bars indicate standard deviations.

Infection intensity was measured in 42 individuals using the faecal sampling method and 43 using the gut dissection method. 5 individuals were lost from the experiment; 3 from the faecal and 2 from the gut sampling group, as they did not drink the inoculum. The best model included method and colony as fixed factors. Bee mass and experimental block did not significantly affect infection intensity (*X*_1_ = 92.969, *P* = 0.256; *X*_1_ = 93.473, *P* = 0.18) and were removed from the final model as they did not improve model fit. In the reduced model, method significantly affected infection intensity (*X*_1_ = 17.993, *P* = <0.001; Figure 1). When the gut dissection method was used to measure infection intensity the mean infection intensity was significantly different to and approximately double (9415 [±1.11] cells/*μ*L), those obtained *via* the faecal sampling method (4915 [±1.11] cells/*μ*L). Colony also significantly affected infection intensity (X_2_ = 9.386, *P* *=* 0.00916; Figure 2). Colony 1 had significantly higher infection intensity across both methods than colony 2 (colony 1: 9228 [±1.14] cells/*μ*L, colony 2: 5377 [±1.14] cells/*μ*L; *P* = 0.0088). Infection intensity was not significantly different in colony 2 compared to colony 3 (colony 2: 5377 [±1.14] cells/*μ*L, colony 3: 6311[±1.14] cells/*μ*L; *P* *=* 0.660) or colony 1 compared to colony 3 (*P* *=* 0.105).

## Discussion

Here, we found that the method used to measure the infection intensity of *C. bombi* in *B. terrestris* affects the estimate of infection intensity. Specifically, the gut sampling method generated measures of infection intensity that were almost double those from the faecal sampling method. Infection intensity was also affected by the colony bees originated from, with colony 1 exhibiting higher infection intensities than colony 2 irrespective of the methods used to measure infection intensity.

The higher estimates of infection intensity found when using the gut as opposed to the faecal sampling method demonstrate that these measures are not equivalent or comparable. However, the direction of difference contrasts with the results from Otterstatter and Thomson (2006), which found higher estimates of infection intensity in faecal compared to gut samples. Interestingly, their faecal estimates were almost double that from the gut, which is a similar magnitude of difference to our results. The difference between our 2 studies is likely driven by the fact that Otterstatter and Thomson (2006) took both measurements from the same individual. By definition, when both approaches are used on the same individual, gut sampling must always be conducted after faecal sampling. Therefore, it is likely that the number of *C. bombi* cells were lower in the gut because the individual had recently defecated and cleared a large number of cells from the gut. In contrast, we used individuals for either faecal or gut sampling, but not both. Consequently, we have removed the possibility of this order effect affecting a comparison of the methods. In our experiment it is possible that individuals defaecated prior to being frozen for dissection. However, firstly it was not possible to control this and secondly, this effect would have been random and the chance of a large number of bees defaecating immediately prior to being frozen is relatively small.

Although the effect of method on estimates of prevalence could not be tested, the higher infection intensity estimates obtained using the gut sampling method suggest that this method is more sensitive and thus more likely to identify infection accurately in animals with low infection intensity. This is an important consideration when choosing which method to use when designing a study. If one is expecting low infection intensity the gut sampling method may be more suitable. The gut sampling method may also be able to detect newly established infections earlier when the parasite has just started replicating in the gut, and cell concentration in the faeces is very low, making it a better measure of prevalence. The choice of method should also be determined by the hypotheses being tested, since one could argue that these methods measure slightly different infection outcomes. The faecal sampling method measures the infectivity of the individual, whereas the gut sampling method measures the parasite load in the gut. Whilst these values are positively correlated within individuals (Otterstatter and Thomson, 2006), our results have emphasized that they cannot be compared as equal measures of *C. bombi* infection outcomes. Time availability during and after the experiment also needs to be considered when choosing a method. The faecal sampling method is more time-sensitive and will impose a limit on the number of samples that can be collected within 1 day (~15–18 per hour in our experience), whereas after the samples are frozen the gut sampling method is less time-sensitive. This is because dissection can be done at any time, however, more time is required per sample to obtain an estimate. Approximately 12–15 min per sample is required to dissect and measure infection using the gut method, compared to approximately 4 min for the faecal method (H.W.G., pers. obs.). Using the faecal sampling method also enables individuals to remain alive and, therefore, can be used if repeated sampling of the same individual is required. Finally, if one is new to measuring *C. bombi* infection the time needed to learn a technique may be a consideration. In our experience both methods are similar in their complexity. For example, when using the faecal sampling method some cells swim fast and can be hard to count, whereas, when using the gut sampling method, learning how to remove the guts and distinguishing cells from gut debris can be challenging.

In addition to informing the designs of future studies, our results are valuable when interpreting the results of previous studies, as higher infection intensities will be expected if the gut sampling method was used to measure infection. When comparing results between studies, the inoculation dose and the settling times vary (see Table S1 and S2). However, variation in these are unlikely to affect results because firstly, inoculation doses above 1250 cells does not affect infection intensity (Schmid-Hempel *et al*., 2019) and secondly, settling time does not affect estimates of infection intensity (Otterstatter and Thomson, 2006). Given these caveats, if infection intensity estimates from previous studies are compared, it is clear that infection intensity estimates vary (see Table S2). However, the gut-method estimates are not approximately double the faecal-method estimate, as our results would predict. This could be because studies generally use 1 method of measuring infection and therefore, direct comparison within 1 study is not possible. Estimates from various studies may not be directly comparable because different hypotheses are being tested, for example, bees may be fed different pollen diets that can affect infection intensity (e.g. Giacomini *et al*., 2018; Fowler *et al*., 2022). Furthermore, in each study different host and parasite genes are interacting, which can affect the infection outcome as this system exhibits host–parasite genotype–genotype interactions (Baer and Schmid-Hempel, 2003; Cisarovsky *et al*., 2012; Barribeau and Schmid-Hempel, 2013). In addition, often laboratory studies that use the gut sampling method use *B. impatiens* as a host (e.g. Anthony *et al*., 2015; Giacomini *et al*., 2018; Aguirre *et al*., 2020; Fowler *et al*., 2022), whereas, studies that use the faecal sampling method often use *B. terrestris* as a host (e.g. Schmid-Hempel *et al*., 1999; Logan *et al*., 2005; Yourth and Schmid-Hempel, 2006; Folly *et al*., 2020) (see Table S2). This confounding use of sampling method and host species makes cross-species comparison of infection estimates challenging. When infection estimates are approximately compared between *B. terrestris* and *B. impatiens* (see Table S2), susceptibility appears not to differ between the species, however, it is not possible to attribute differences in infection estimates to species due to the confounding use of sampling method and host species.

In addition, studies use different methods to count *C. bombi* cells, which may also affect infection estimates. For example, some studies count ‘live’ or ‘actively moving’ *C. bombi* cells (LoCascio, Pasquale, *et al*., 2019*b*; Aguirre *et al*., 2020). The criteria for which cells are being counted are not explicitly specified in these studies, but ‘live’ or ‘actively moving’ likely refers to only 2 of the cell types of this parasite (choanomastigote and promastigote, both of which have flagella and swim actively). Given that amastigotes are common and abundant across the timeline of infections (Logan *et al*., 2005), such criteria likely leads to lower intensity estimates when compared to studies that count all of the cell types. Indeed, estimates in these studies are relatively low, for example, LoCascio, Pasquale, *et al*. (2019*b*) measured 1750 cells/*μ*L 7 days post-inoculation following a 6000 cell dose (as detailed above) and Aguirre *et al*. (2020) estimated infection intensities of 55.5–750 cells/*μ*L 7 days post-inoculation with a 6000 cell dose.

Another significant predictor of infection intensity was colony. On average, 1 colony consistently exhibited higher infection intensities irrespective of methods. This is a well-established effect in the system, as the host-parasite interaction exhibits genotype–genotype specificity, with some *C. bombi* strains more likely to infect certain colony genotypes (Baer and Schmid-Hempel, 2003; Cisarovsky *et al*., 2012). In addition, host colonies exhibit a range of immune gene expression, which may affect their susceptibility to infection (Schlüns *et al*., 2010; Brunner *et al*., 2013). Furthermore, susceptibility varies between colonies due to differences in the gut microbiome. Similar to host genotype, the host microbiome can have a large effect on infection intensity (Koch and Schmid-Hempel, 2012; Mockler *et al*., 2018). These 2 factors are linked as genotype can influence which microbiota establish in the gut (Koch and Schmid-Hempel, 2012). Consequently, some colonies are more susceptible to *C. bombi* infection, as seen in our experiment. Ecologically, this means that some colonies are likely to take the role of super spreaders in driving the annual parasite epidemic.

In conclusion, using the gut sampling method to measure infection intensity, following a standardized inoculation dose, produced cell counts that were almost double those from the faecal sampling method. Our results have implications for the design of future studies, as if low levels of infection are expected or if sampling early in the infection, the gut sampling method may be a more sensitive method of measuring infection. However, the faecal method provides a more accurate estimation of the infectiousness of an individual and is more suitable if it is necessary for bees to remain alive. Further considerations for choosing a method include the time available during an experiment and the learning required to conduct each sampling method effectively. Our results emphasize that an understanding of how results from different methodologies vary can be valuable in the interpretation of results from previous studies. In conclusion, given the importance of bumblebees as pollinators, the impact of *C. bombi* on bumblebee health, and its use as an epidemiological model, we call on researchers to move towards consistent quantification of infections to enable future comparisons and meta-analyses of studies.

## Supporting information

Wolmuth-Gordon et al. supplementary material Wolmuth-Gordon et al. supplementary material

## Data Availability

Data available at: https://doi.org/10.5281/zenodo.10118532

## Supplementary material

The supplementary material for this article can be found at https://doi.org/10.1017/S0031182023001002
