# Supplementary material for: Methods matter: the influence of method on infection estimates of the bumblebee parasite Crithidia bombi
Source: Parasitology. 2023 Oct 20;150(13):1236–41. doi: 10.1017/S0031182023001002 (PMC10941228; doi:10.1017/S0031182023001002)
Supplement: Wolmuth-Gordon et al. supplementary material [file S0031182023001002sup001.docx]

Supplementary material

**Table S1:** Comparison of gut methods used in previous studies.

| **Paper** | **Settling time (hrs)** | **Gut dissected** | **Grinding guts** | **Other notes** |
| --- | --- | --- | --- | --- |
| Anthony, W. E., Palmer-Young, E. C., Leonard, A. S., Irwin, R. E., & Adler, L. S. (2015).  Testing dose-dependent effects of the nectar alkaloid anabasine on trypanosome parasite loads in adult bumble bees. *PLoS ONE*. https://doi.org/10.1371/journal.pone.0142496 | 3-8 | Mid and hindguts not dissected frozen | Ground guts in 300ul distilled water |  |
| Biller, O. M., Adler, L. S., Irwin, R. E., McAllister, C., & Palmer-Young, E. C. (2015).  Possible synergistic effects of thymol and nicotine against Crithidia bombi parasitism in bumble bees. *PLoS ONE*. https://doi.org/10.1371/journal.pone.0144668 | 4 | Gut tracts not frozen | Ground guts in 300ul distilled water |  |
| Palmer-Young, E. C., Hogeboom, A., Kaye, A. J., Donnelly, D., Andicoechea, J., Connon, S. J., Weston, I., Skyrm, K., Irwin, R. E., & Adler, L. S. (2017).  Context-dependent medicinal effects of anabasine and infection-dependent toxicity in bumble bees. *PLoS ONE*. https://doi.org/10.1371/journal.pone.0183729 | 5 | Intestinal tracts not frozen | Ground guts 300ul distilled water |  |
| Giacomini, J. J., Leslie, J., Tarpy, D. R., Palmer-Young, E. C., Irwin, R. E., & Adler, L. S. (2018).  Medicinal value of sunflower pollen against bee pathogens. *Scientific Reports*. https://doi.org/10.1038/s41598-018-32681-y | 4-5 | Digestive tracts excluding honey crop not frozen | Ground guts in 300ul 25% strength Ringer’s solution |  |
| LoCascio, G. M., Pasquale, R., Amponsah, E., Irwin, R. E., & Adler, L. S. (2019).  Effect of timing and exposure of sunflower pollen on a common gut pathogen of bumble bees. *Ecological Entomology*. https://doi.org/10.1111/een.12751 | 3-4 | Guts (not specified) not frozen | Ground guts with 300ul 25% Ringer’s solution | Counted ‘live and actively moving Crithidia’ |
| LoCascio, G. M., Aguirre, L., Irwin, R. E., & Adler, L. S. (2019).  Pollen from multiple sunflower cultivars and species reduces a common bumblebee gut pathogen. *Royal Society Open Science*. https://doi.org/10.1098/rsos.190279 | 4-5 | Mid and hindguts not frozen | Ground guts in 300ul of 25% Ringer’s solution |  |
| Aguirre, L. A., Davis, J. K., Stevenson, P. C., & Adler, L. S. (2020).  Herbivory and Time Since Flowering Shape Floral Rewards and Pollinator-Pathogen Interactions. *Journal of Chemical Ecology*. https://doi.org/10.1007/s10886-020-01213-2 | 4 | Hindguts not frozen | Ground hindguts in Ringer’s solution | Counted ‘live’ Crithidia cells |


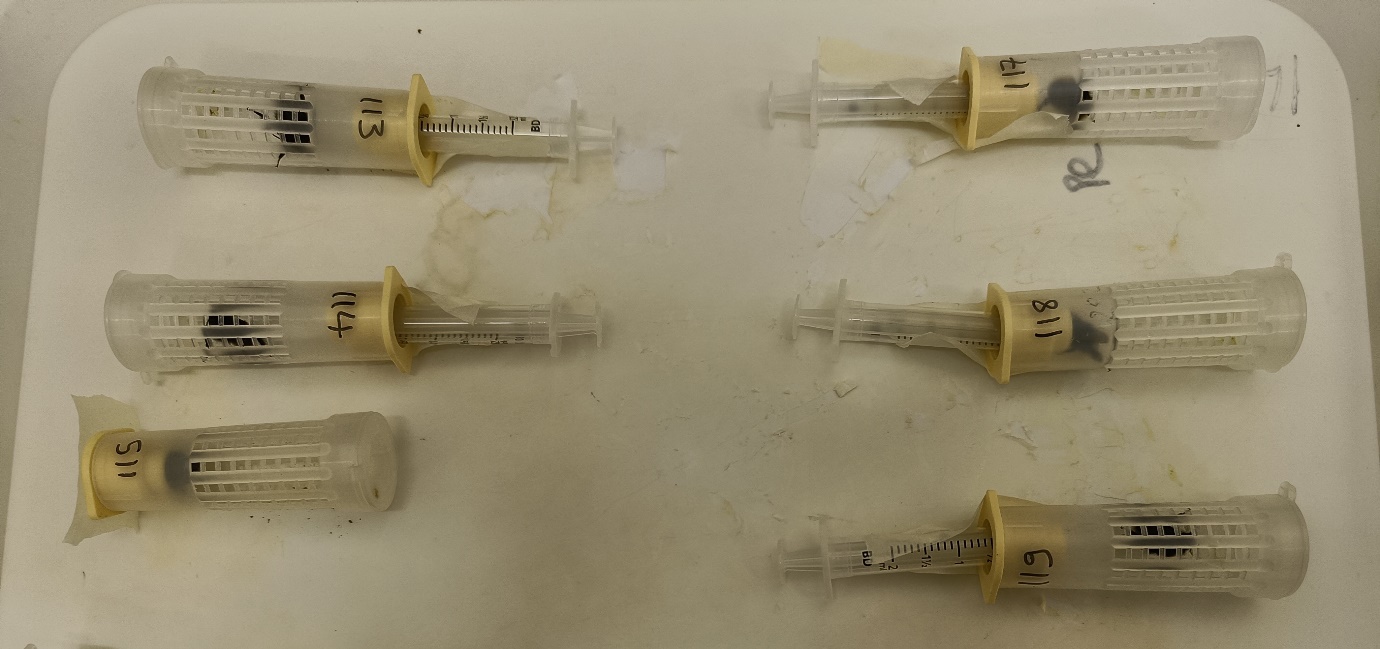


**Fig. S1** *Bombus terrestris audax* were housed in nicot cages (Becky’s bees, UK) throughout the experiment. Syringes were taped to the base of the nicot cage and used to inoculate and feed the bees.

**Table S2.** Comparison infection prevalence and intensity estimates from studies that use the gut and faecal sampling method. When empirical data was not available, Webplot digitizer was used to extract data from figures.

| Sampling method | Study | Host species | Dose (cells) | Days post-infection | Infection intensity (cells/μL) | Infection prevalence (%) | Notes |
| --- | --- | --- | --- | --- | --- | --- | --- |
| Gut | Aguirre LA, Davis JK, Stevenson PC, Adler LS. Herbivory and Time Since Flowering Shape Floral Rewards and Pollinator-Pathogen Interactions. J Chem Ecol. 2020;46:978–986. doi:10.1007/s10886-020-01213-2 | *B. impatiens* | 6,000 | 7 | 555 |  | Counted ‘live’ cells  Estimate taken from ‘within 1 month undamaged’ control treatment. |
|  | Fowler AE, Giacomini JJ, Connon SJ, Irwin RE, Adler LS. Sunflower pollen reduces a gut pathogen in the model bee species, Bombus impatiens, but has weaker effects in three wild congeners. Proc R Soc B Biol Sci. 2022;289(1968):20211909. doi:10.1098/rspb.2021.1909 | *B. impatiens* | 6,000 | 35 | wild colony 2016: 14,450  commercial colony 2016: 2,250  wild colony 2020: 2,600 | 100  88  93 | Estimate taken from buckwheat control treatment. |
|  | Giacomini JJ, Leslie J, Tarpy DR, Palmer-Young EC, Irwin RE, Adler LS. Medicinal value of sunflower pollen against bee pathogens. Sci Rep. 2018;8:14394. doi:10.1038/s41598-018-32681-y | *B. impatiens* | 6,000 | 7 | Buckwheat pollen treatment: 5,500  Rapeseed pollen treatment: 2,000 |  | Estimate taken from buckwheat and rapeseed control treatments. |
|  | Anthony WE, Palmer-Young EC, Leonard AS, Irwin RE, Adler LS. Testing dose-dependent effects of the nectar alkaloid anabasine on trypanosome parasite loads in adult bumble bees. PLoS One. 2015;10(11):e0142496. doi:10.1371/journal.pone.0142496 | *B. impatiens* | 6,000 | Upon death | 885 | 70 | Estimate taken from 0ppm anabasine treatment. |
| Faecal | Schmid-Hempel P, Puhr K, Krüger N, Reber C, Schmid-Hempel R. Dynamic and genetic consequences of variation in horizontal transmission for a microparasitic infection. Evolution (N Y). 1999;53(2):426-434. doi:10.1111/j.1558-5646.1999.tb03778.x | *B. terrestris* | 10,000 | 6 | 4,000 |  | Estimate taken from ‘early, immediate’ treatment group |
|  | Yourth CP, Schmid-Hempel P. Serial passage of the parasite Crithidia bombi within a colony of its host, Bombus terrestris, reduces success in unrelated hosts. Proc R Soc B Biol Sci. 2006;273:655-659. doi:10.1098/rspb.2005.3371 | *B. terrestris* and *B. lucorum* | 10,000 | 7 |  | 71 | Estimate taken from single passage between unrelated colonies. |
|  | Logan A, Ruiz-González MX, Brown MJF. The impact of host starvation on parasite development and population dynamics in an intestinal trypanosome parasite of bumble bees. Parasitology. 2005;130(6):637-642. doi:10.1017/S0031182005007304 | *B. terrestris* | 25,000 | 7 | 27,600 |  | Estimate taken from pollen treatment group. |
|  | Folly AJ, Barton-Navarro M, Brown MJF. Exposure to nectar-realistic sugar concentrations negatively impacts the ability of the trypanosome parasite (Crithidia bombi) to infect its bumblebee host. Ecol Entomol. 2020;45(6):1495-1498. doi:10.1111/een.12901 | *B. terrestris* | 10,000 | 7 | Sugar conc. treatments:  10%: 5,922  20%: 7,608  40%: 12,835  60%: 4,350 | 69  64  55  71 | Estimates taken from four sugar concentrations. |
